# Effects of Tracer Uptake Time in Non–Small Cell Lung Cancer ^18^F-FDG PET Radiomics

**DOI:** 10.2967/jnumed.121.262660

**Published:** 2022-06

**Authors:** Guilherme D. Kolinger, David Vállez García, Gerbrand Maria Kramer, Virginie Frings, Gerben J.C. Zwezerijnen, Egbert F. Smit, Adrianus Johannes de Langen, Irène Buvat, Ronald Boellaard

**Affiliations:** 1 Medical Imaging Center, University Medical Center Groningen, University of Groningen, Groningen, The Netherlands;; 2 Department of Radiology and Nuclear Medicine, Amsterdam University Medical Center, VU Medical Center, Amsterdam, The Netherlands;; 3 Department of Pulmonology, Amsterdam University Medical Center, VU Medical Center, Amsterdam, The Netherlands;; 4 Department of Thoracic Oncology, Antoni van Leeuwenhoek Hospital, Amsterdam, The Netherlands; and; 5 Laboratoire d’Imagerie Translationnelle en Oncologie, INSERM, Institut Curie, Université Paris-Saclay, Orsay, France

**Keywords:** PET, radiomics, texture analysis, repeatability, dual-time-point

## Abstract

PET radiomics applied to oncology allow the measurement of intratumoral heterogeneity. This quantification can be affected by image protocols; hence, there is an increased interest in understanding how radiomic expression on PET images is affected by different imaging conditions. To address that interest, this study explored how radiomic features are affected by changes in ^18^F-FDG uptake time, image reconstruction, lesion delineation, and radiomic binning settings. **Methods:** Ten non–small cell lung cancer patients underwent ^18^F-FDG PET on 2 consecutive days. On each day, scans were obtained at 60 and 90 min after injection and reconstructed following EARL version 1 and with point-spread-function resolution modeling (PSF-EARL2). Lesions were delineated with an SUV threshold of 4.0, with 40% of SUV_max_, and with a contrast-based isocontour. PET image intensity was discretized with both a fixed bin width (FBW) and a fixed bin number before the calculation of the radiomic features. Repeatability of features was measured with the intraclass correlation coefficient, and the change in feature value over time was calculated as a function of its repeatability. Features were then classified into use-case scenarios based on their repeatability and susceptibility to tracer uptake time. **Results:** With PSF-EARL2 reconstruction, 40% of SUV_max_ lesion delineation, and FBW intensity discretization, most features (94%) were repeatable at both uptake times (intraclass correlation coefficient > 0.9), 35% being classified for dual-time-point use cases as being sensitive to changes in uptake time, 39% were classified for cross-sectional studies with an unclear dependency on time, 20% were classified for cross-sectional use while being robust to uptake time changes, and 6% were discarded for poor repeatability. EARL version 1 images had 1 fewer repeatable feature (neighborhood gray-level different matrix coarseness) than PSF-EARL2; the contrast-based delineation had the poorest repeatability of the delineation methods, with 45% of features being discarded; and fixed bin number resulted in lower repeatability than FBW (45% and 6% of features were discarded, respectively). **Conclusion:** Repeatability was maximized with PSF-EARL2 reconstruction, lesion delineation at 40% of SUV_max_, and FBW intensity discretization. On the basis of their susceptibility to uptake time, radiomic features were classified into specific non–small cell lung cancer PET radiomics use cases.

## Visual Abstract

**Figure:**
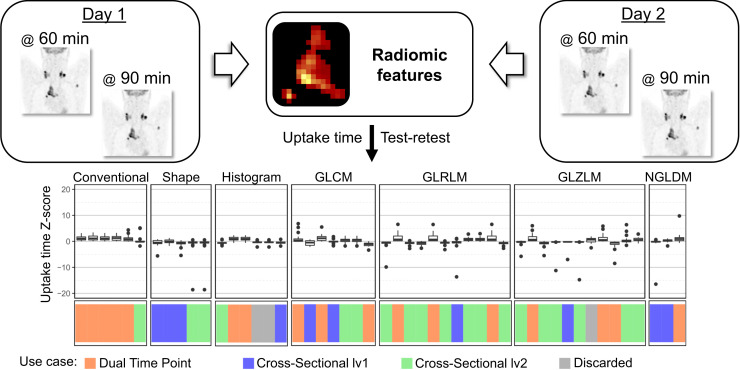

For staging and treatment response evaluation of patients with non–small cell lung cancer (NSCLC), ^18^F-FDG PET/CT is an important technique. This evaluation can be achieved either visually or using SUVs and total lesion glycolysis measurements (1–5). However, these semiquantitative approaches ignore possible tracer uptake heterogeneity within the tumor (6), overlooking potentially useful information. To address this issue, the field of Radiomics has been developed to perform measurements of textural information available in medical images, resulting in a more complete phenotyping of the tumor (7–9).

PET radiomics in oncology allow the extraction of several features characterizing tumor uptake, shape, and intratumoral heterogeneity (10–13). This approach showed promising results, including lesion histological sub-type identification, aided automated tumor delineation and disease-free survival prediction (14–18). Despite this success, radiomic features are sensitive to image noise, lesion segmentation method, signal intensity discretization, and several image settings, including PET acquisition and reconstruction settings (11*,*^19^–^26^). This sensitivity leads to difficulties in multicenter studies, possibly explaining why the results have poor reproducibility, leading to skepticism about the usefulness of radiomics (9*,*^19^*,*^27^–^31^). Furthermore, these issues are amplified by the lack of negative publications on the field (32). Strategies have been developed to mitigate this variability and thus improve the postreconstruction harmonization of textural features (33–35).

One aspect of ^18^F-FDG PET radiomics that has not been extensively explored is its uptake time dependence. The time between tracer injection and image acquisition alters the uptake in metabolically active regions where ^18^F-FDG gradually accumulates, affecting SUV-related metrics and their repeatability (36–38). ^18^F-FDG PET/CT textural analysis from dual-time-point static scans has been used to differentiate benign from malignant pulmonary lesions despite features presenting a wide range of accuracy (39*,*^40^). Time-related PET radiomics have been also explored as dynamic features (41). However, neither of these studies assessed how uptake time could influence textural feature repeatability.

Our hypothesis was that different features have different levels of dependence on uptake time and that this dependence may be influenced by image settings. Therefore, we evaluated how radiomic features (SUV-based and textural) are affected by uptake time and whether its effects are smaller or larger than the effects of feature repeatability. On the basis of each feature’s repeatability and dependence on uptake time, features are classified into cross-sectional or single-injection dual-time-point use cases. Several image settings are considered, including PET/CT image reconstruction algorithms, lesion delineation methods, and intensity discretization strategies.

## MATERIALS AND METHODS

### Dataset

Ten patients with confirmed stage IIIB or IV NSCLC underwent double baseline ^18^F-FDG PET/CT on a Gemini TF scanner (Philips Healthcare), as previously described (5*,*^20^). Patients fasted for 6 h or more, and then a low-dose CT scan was acquired for attenuation correction followed by a whole-body ^18^F-FDG PET scan 60 min after tracer injection. Thirty minutes later, a second whole-body PET scan and low-dose CT scan were obtained. This procedure was repeated within 3 d of the first scan for test–retest measurements. All PET data were normalized and corrected for scatter and random events, dead time, attenuation, and decay. Two reconstruction protocols were used, one following the EARL version 1 guidelines (EARL1) and another with point-spread-function resolution modeling (PSF-EARL2) (42–44). The PET images had a final resolution of 144 × 144 × 254 voxels with a voxel size of 4 × 4 × 4 mm^3^. The average injected activity was 248 MBq (range, 194–377 MBq) on the first day and 238 MBq (range, 192–392 MBq) on the second day. The average postinjection start times were 61 min (range, 59–67 min) and 92 min (range, 90–97 min) on the first day and 60 min (range, 60–63 min) and 90 min (range, 90–95 min) on the second day. All patients gave written informed consent before enrollment, and the study was approved by the Medical Ethics Review Committee of the Vrije Universiteit Medical Center (Dutch trial register NTR3508; https://www.trialregister.nl/).

### Radiomic Feature Extraction

Lesions were delineated and radiomic features extracted using LIFEx (version 6.30) (45). All lesions were included for the analysis, namely the primary and metastatic lesions (intra- and extrathoracic), yielding 1–10 lesions as a function of the patient. Lesions were delineated on the PSF-EARL2 PET images using an isocontour at 40% of each lesion’s SUV_max_, and then radiomic features were extracted with intensity discretization using a fixed bin width (FBW) of 0.25 g/mL, ranging from 0–60 g/mL for each lesion (the 60 g/mL upper bound was higher than the SUV_max_ of all lesions). This combination of image and processing settings was considered the reference settings for radiomic analysis, as they were previously shown to optimize test–retest variability (19*,*^36^*,*^46^). Other image settings were explored, including lesion delineation and feature extraction from EARL1 images, lesion delineation with a fixed isocontour at an SUV threshold of 4.0 (SUV4) and a contrast-based isocontour at 0.5 × SUV_peak_ + background SUV (contrast; background SUV was the mean uptake in a shell 2 cm away from the volume defined at 70% of SUV_max_, excluding voxels with SUV > 4), and intensity discretization with a fixed bin number (FBN) of 64 bins in a variable range of SUV_min_–SUV_max_.

In total, 49 radiomic features from 7 classes were extracted (the full list is given in Supplemental Table 1; supplemental materials are available at http://jnm.snmjournals.org): 6 conventional PET metrics, 5 shape-based features, 6 histogram-based features, 7 gray-level cooccurrence matrix (GLCM) features, 11 gray-level run-length matrix (GLRLM) features, 11 gray-level zone-length matrix (GLZLM) features, and 3 neighborhood gray-level difference matrix (NGLDM) features. Features were obtained only for lesions that included at least 64 voxels. LIFEx’s feature definition is in compliance with the Image Biomarker Standardisation Initiative (47*,*^48^).

### Data Analysis

Features calculated from images obtained at different time points on the first day of scans were statistically compared using pairwise Wilcoxon signed-rank tests. *P* values below 0.05 were considered statistically significant after Benjamini–Hochberg false-discovery-rate correction. A change in feature value was measured as a function of uptake time by using its test–retest variability at 60 min after injection as a baseline (analogous to a *z* score):

$$z=\frac{\left({\text{RF}}_{90}-{\text{RF}}_{60}\right)-{\text{mean}\;\text{TRT}}_{60}}{{\text{TRT}}_{60}\;\text{SD}\;}.$$

RF_60_ and RF_90_ represent the radiomic feature values at 60 and 90 min after injection, respectively. TRT_60_ is the test–retest difference between the feature values at the second- and first-day scans (at 60 min after injection). Therefore, the effects of uptake time on radiomic features were contextualized with respect to repeatability: *z* scores lower than 1 indicate changes with an uptake time less than test–retest variability, and *z* scores higher than 1 show a change larger than repeatability.

**FIGURE 1.**
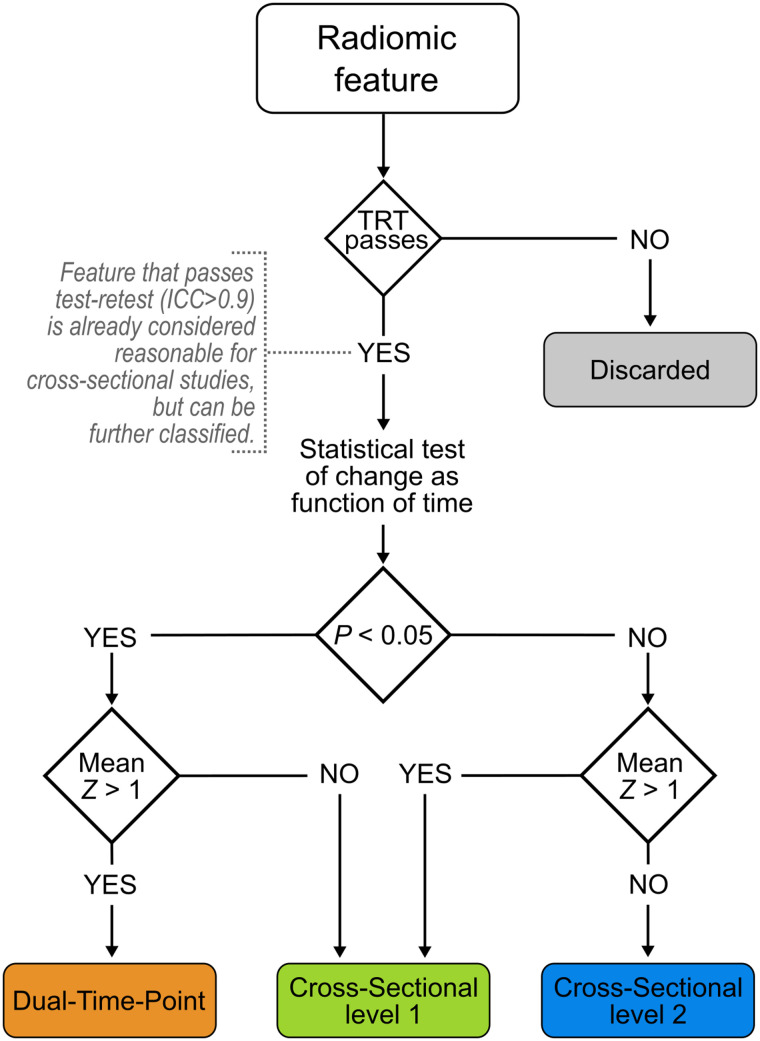
Flowchart for use-case classification of radiomic features. TRT = test–retest; ICC = intraclass correlation coefficient.

A feature was considered repeatable if the intraclass correlation coefficient (agreement type, 2-way mixed-effects model, single rating) between test and retest scans (same reconstruction, delineation method, and discretization) was higher than 0.9 at both time points. A feature was defined as robust against change in uptake time if it was not significantly affected by uptake time after false-discovery-rate correction and if its change from 60 to 90 min was less than from one day to another (i.e., mean *z* score < 1). Finally, features were assigned to a use case on the basis of their repeatability and susceptibility to uptake time (Fig. 1): features that were repeatable and susceptible to uptake time were classified for dual-time-point studies, repeatable features with an uncertain response to uptake time were classified as cross-sectional level 1 (CS1), repeatable features that were robust to uptake time were classified as cross-sectional level 2 (CS2), and features with poor repeatability at any time point were discarded. Statistical analysis was done using R, version 4.0.4.

## RESULTS

### Feature Dependence on Uptake Time

**FIGURE 2.**
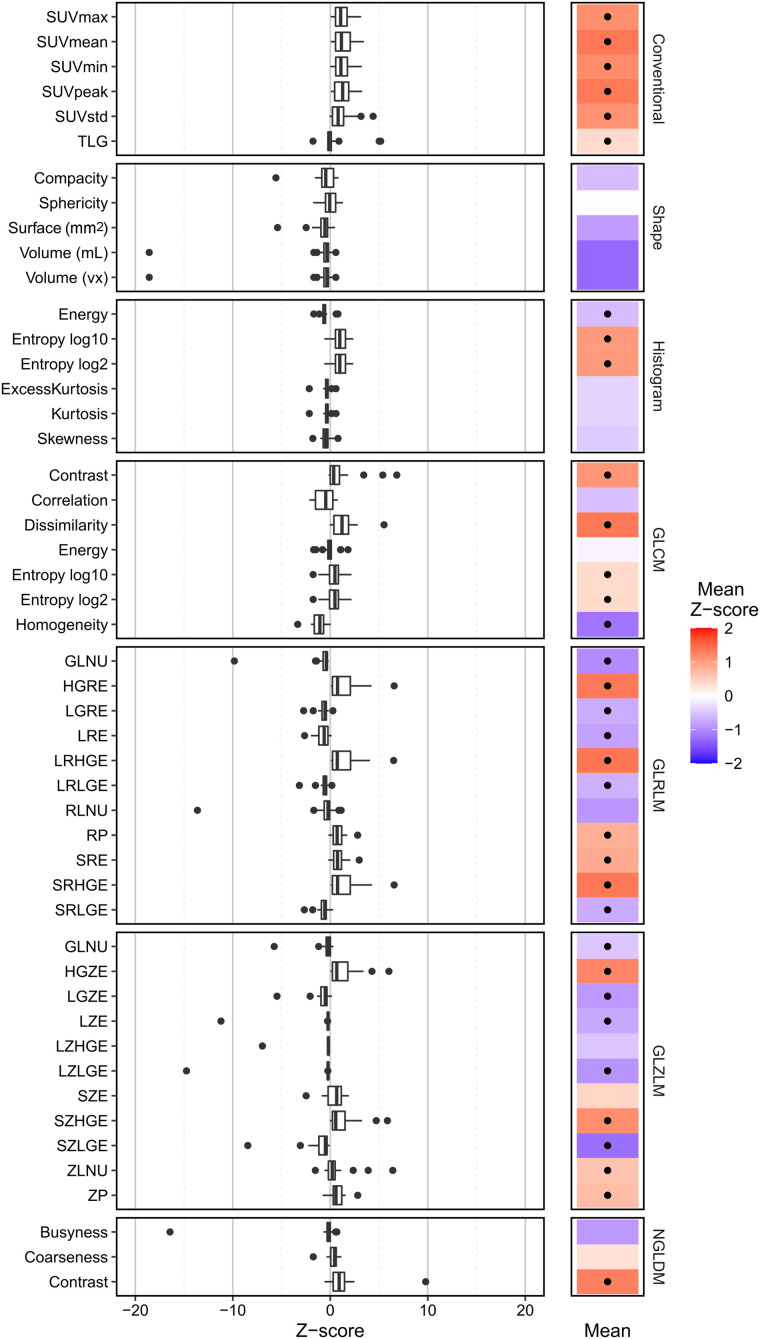
Effect of uptake time on radiomic features. (Left) Distribution of *z* scores for each feature. *z* scores were calculated using test–retest variability on scan at 60 min after injection as baseline. (Right) Mean *z* score of each feature (dot indicates statistical significance). Analysis was of images with reference settings. Abbreviations are defined in Supplemental Table 1.

All conventional features were significantly affected by uptake interval and increased in value with increased uptake time (Fig. 2, positive mean *z* score). Shape features did not significantly differ between the 2 uptake times. Half the histogram features were affected by uptake time (histogram entropy log10 and histogram entropy log2 are equivalent after rescaling with *z* scores). Four of 7 GLCM features significantly increased over time, and only 1 decreased. One GLRLM, 2 GLZLM, and 2 NGLDM features were not statistically significantly dependent on uptake time (Fig. 2). The features of each class with the highest *z* score and a statistically significant (*P* < 0.05) dependence on uptake time were conventional SUV_mean_, histogram entropy, GLCM dissimilarity, GLRLM long-run high-gray-level emphasis, GLZLM short-zone low-gray-level emphasis, and NGLDM contrast (average *z* score ± SD: 1.36 ± 0.98, 1.04 ± 0.73, 1.35 ± 1.29, 1.38 ± 1.69, −1.24 ± 1.86, and 1.28 ± 2.10, respectively).

### Radiomic Feature Use-Case Classification

**FIGURE 3.**
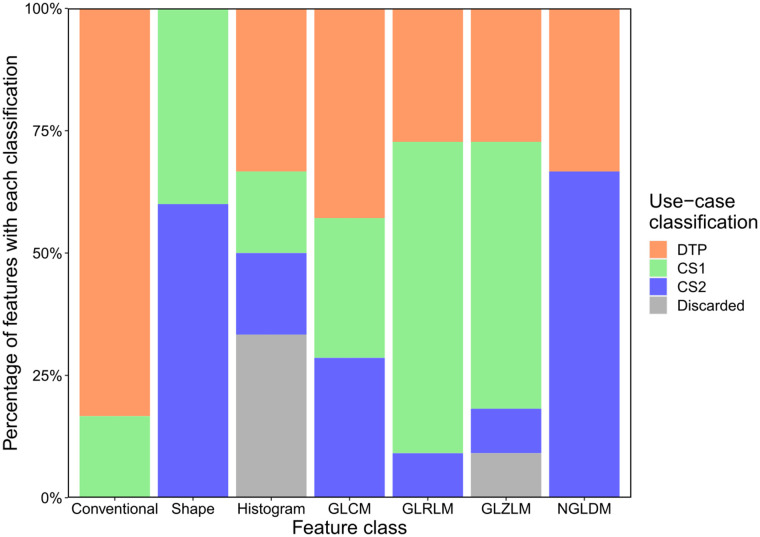
Percentage of radiomic features with each use-case classification for each feature class. Analysis was of images with reference settings. DTP = dual time point.

Ninety-four percent (46/49) of the features had reliable repeatability (intraclass correlation coefficient > 0.9, Supplemental Fig. 1). In total, 35% (17/49) of features were classified as dual-time-point, 39% (19/49) as CS1, and 20% (10/49) as CS2; 6% (3/49) were discarded (Supplemental Fig. 1). No conventional feature was classified for CS2 use cases, no shape feature was classified for dual-time-point use cases, and no NGLDM feature was classified for CS1 use cases. The remaining feature classes had a mixed use-case classification (Fig. 3).

### Influence of Image Settings on Repeatability and Use-Case Classification

**FIGURE 4.**
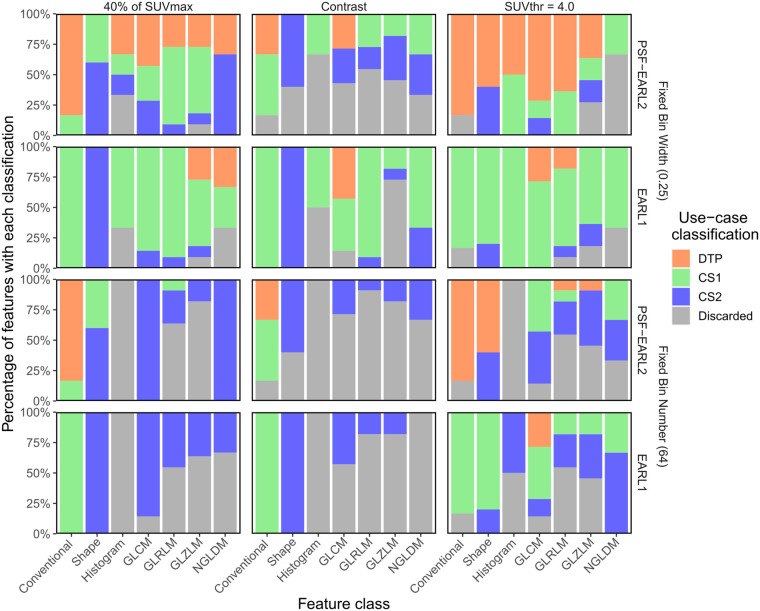
Percentage of radiomic features with each use-case classification for each feature class in all image setting configurations. Columns of panels show different lesion delineation methods, and rows show different image reconstructions and intensity discretization strategies. Analysis was of images with reference settings. DTP = dual time point; SUVthr = SUV threshold.

The reference settings (PSF-EARL2 reconstruction, 40% of SUV_max_ delineation, and FBW discretization) had fewer discarded features than did other image settings (Fig. 4). Images had 1 fewer repeatable feature (NGLDM coarseness) with EARL1 (and recommended delineation and discretization) than with PSF-EARL2 (Fig. 4). With PSF-EARL2 and FBW discretization, the contrast-based lesion delineation method had poorer repeatability than the other methods, and SUV4 had fewer repeatable features than 40% of SUV_max_ (22, 6, and 3 features discarded, respectively). Lastly, repeatability was considerably lower for FBN than for FBW (22 and 3 discarded features with recommended reconstruction and delineation, respectively; Supplemental Fig. 2).

Using the reference delineation and discretization, EARL1 had no conventional feature classified for dual-time-point use cases (Fig. 4; Supplemental Fig. 3). Histogram features were classified only for CS1 use cases (or were discarded), whereas all shape features were classified for CS2. In total, 8% (4/49) of features had a dual-time-point classification, 67% (33/49) had CS1, 16% (8/49) had CS2, and 8% (4/49) were discarded with EARL1 reconstruction when using the reference delineation and discretization.

Despite using the reference reconstruction and discretization, the contrast-based delineation approach resulted in 45% (22/49) of features being discarded (Fig. 4; Supplemental Fig. 3). With SUV4, 12% (6/49) of features were discarded, and all repeatable conventional features had a dual-time-point classification; other feature classes had mixed use-case classifications.

Using FBN for discretization resulted in use-case classifications different from those of FBW, even when both used the reference reconstruction and delineation methods (Fig. 4). The exceptions were the conventional and shape features, since those are not dependent on the image intensity discretization (Supplemental Fig. 3). With PSF-EARL2, 40% of SUV_max_, and FBN, only 1 gray-level–based feature was classified for CS1: GLRLM run length nonuniformity. Furthermore, all GLCM and NGLDM features were robust to uptake time with FBN discretization (CS2 use cases), and all histogram features were discarded (Fig. 4).

## DISCUSSION

This study demonstrated that for PET images reconstructed with PSF-EARL2, lesion delineation with 40% of SUV_max_, and intensity discretization using FBW, most (94%) traditional and gray-level–based features were repeatable on scans at both 60 and 90 min after injection. From the radiomic features assessed, 35% were repeatable and able to detect a change as a function of uptake time (dual-time-point), 39% were repeatable but had an unclear dependency on uptake time (CS1), 20% were repeatable and robust against uptake time changes (CS2), and 6% were not repeatable (discarded). Additionally, analyses performed on PET images reconstructed using EARL1, lesion delineation using a contrast-based approach or a fixed threshold method, and intensity discretization using a fixed number of bins decreased repeatability and led to different use-case classifications of radiomic features.

Overall, more features significantly increased (22/49) with time than decreased (12/49), as found previously (49). Conventional features increased over time, as expected (50*,*^51^), and shape features slightly decreased in the delayed PET scan. This decrease in volume due to a higher threshold for lesion delineation (at 40% of SUV_max_) agrees with the lower metabolic tumor volume of breast cancer for delayed PET scans (52). The statistically significant histogram features affected by uptake time were energy (decreased) and entropy (increased). The first is related to the uniformity of the distribution and the second to its randomness, therefore reflecting an increase in tumor heterogeneity on delayed ^18^F-FDG PET scans (52). Yet, these features were not significantly affected by uptake time on peripheral nerve sheath tumors with a relatively low ^18^F-FDG uptake (49), emphasizing that translation of radiomic results between different tumor types must be performed with caution even with first-order features.

The increase in GLRLM run percentage, GLZLM zone percentage, and NGLDM contrast over time reflects an increased heterogeneity, as run percentage and zone percentage are low for highly uniform volumes of interest (47) and contrast is related to the intensity difference between neighboring regions. However, there was a decrease in GLRLM and GLZLM nonuniformity, suggesting a reduction in heterogeneity over time. These nonuniformity features have previously been reported as being dependent on time (49*,*^52^), but with a small effect size and a direction of change that was not uniform across studies. Therefore, more features suggest an increase in tumor heterogeneity over time than a decrease, agreeing with previous findings for advanced breast cancer (52) but disagreeing with peripheral nerve sheath tumor results (49). This incompatibility may come from the uptake levels in the tumors. The present study and Garcia-Vicente et al. (52) assessed tumors with relatively high ^18^F-FDG uptake and found increasing heterogeneity over time, whereas Lovat et al. (49) studied low-uptake lesions.

Radiomic features classified for CS1 use cases were repeatable at both uptake times but did not have any clear relationship with uptake time—that is, were neither robust nor sensitive. These features may be suitable for cross-sectional studies if all images are acquired with similar postinjection times. The dependence of the CS1 features on time could explain some of their variability and range previously found on lung cancer assessment (15*,*^25^*,*^46^). Other repeatable features were robust against changes in uptake time (CS2) and are recommended for studies with an inconsistent postinjection scanning time. In contrast, repeatable features statistically significantly and substantially affected by uptake time were classified for dual-time-point use cases. Like CS1 features, dual-time-point features may be used on images acquired with a similar uptake time (e.g., SUV_mean_) but can also measure the effect of time on feature values. Previous studies have reported a possible added benefit of a dual-time-point scanning protocol for differentiation between benign and malignant pulmonary lesions with textural features (39*,*^40^) and for breast cancer intratumoral heterogeneity assessment (52). Unfortunately, given the different nature of the lesions and analysis settings in those previous studies, it is not possible to directly compare the radiomic features found useful by those authors with the ones we identified as appropriate for dual-time-point studies.

As shown previously (19), EARL1 reconstructions resulted in worse repeatability than PSF-EARL2. Additionally, PSF-EARL2 reconstructions also displayed higher heterogeneity (20) and are recommended for textural analysis. Concerning the lesion delineation method, a fixed isocontour lesion delineation (SUV4) yielded poorer repeatability than an adaptive threshold based on 40% of SUV_max_, as expected from the literature (36). The contrast-based delineation had the poorest repeatability of all methods and is thus not recommended for radiomics. Furthermore, previous findings that the repeatability of FBW intensity discretization is superior to that of FBN for PET radiomics were reproduced (19*,*^46^*,*^47^). In historical cohorts for which only EARL1 reconstruction is available, few features are viable for dual-time-point studies (Fig. 4). With lesion delineation at 40% of SUV_max_ and discretization with FBW, the EARL1 protocol still provides several repeatable radiomic features.

The analysis of data from a single scanner vendor and the inclusion of a single tumor type (NSCLC, including intra- and extrathoracic lesions), especially given that features have different levels of expression for different cancer types, are some limitations of our study, and multicenter studies are needed to verify our findings. Furthermore, voxel size affects radiomic feature values and lesion delineation. However, the impact of voxel size on feature use-case classification still needs to be explored. Data from static scans 30 min apart were evaluated. Nevertheless, it is possible that additional radiomic information could be obtained from scans acquired farther apart in uptake time. Finally, several features were analyzed under different image conditions on only 10 subjects. This study may thus be subject to type 1 errors although a false-discovery-rate correction was applied to the statistical analysis.

In summary, EARL1 reconstruction led to classification of fewer features for dual-time-point use cases than did PSF-EARL2. Textural features were not robust against changes in uptake interval when SUV4 was used for lesion delineation, showing that for NSCLC radiomics, this method should be applied only to PET images acquired with a similar uptake time. Furthermore, most features were discarded when the contrast-based delineation method or the FBN intensity discretization was used, and their use is not recommended for NSCLC ^18^F-FDG PET radiomic studies.

## CONCLUSION

This study demonstrated that PET radiomics can be repeatable, summarized the features’ susceptibility to postinjection PET scanning time, and classified the features into reliable use cases for NSCLC radiomics: dual-time-point and cross-sectional studies. Repeatability and the use case of radiomic features depended on PET image reconstruction, lesion delineation, and intensity discretization, and recommendations were provided accordingly.

## DISCLOSURE

This project received funding from the European Union’s Horizon 2020 research and innovation program under the Marie Skłodowska-Curie Innovative Training Network (grant agreement 764458). Irène Buvat is involved in the development of LIFEx. No other potential conflict of interest relevant to this article was reported.

## Notes

KEY POINTS **QUESTION:** Is the change in radiomic features with ^18^F-FDG uptake time larger than their repeatability, and can that change be used for temporal textural analysis? **PERTINENT FINDINGS:** PET image reconstruction with point-spread-function modeling, lesion delineation at 40% of SUV_max_, and intensity discretization with FBW resulted in repeatable radiomic features on scans at 60 and 90 min after injection and provided reliable information for cross-sectional and dual-time-point studies. **IMPLICATIONS FOR PATIENT CARE:** Radiomic features were identified and classified for potential use cases in cross-sectional and dual-time-point protocols, providing reliable information about tumor heterogeneity for NSCLC assessment.
